# Multiple mechanisms can disrupt oncogenic pathways in multiple myeloma

**DOI:** 10.18632/oncotarget.26301

**Published:** 2018-11-09

**Authors:** Phuc H. Hoang, Richard S. Houlston

**Affiliations:** Division of Genetics and Epidemiology, The Institute of Cancer Research, London, UK; Division of Molecular Pathology, The Institute of Cancer Research, London, UK

**Keywords:** multiple myeloma, cancer genetics, non-coding drivers, coding drivers, integrated analysis

Multiple myeloma (MM) is a clinically and biologically heterogeneous malignancy resulting from the infiltration of clonal plasma cells in the bone marrow [1]. Thus far the molecular mechanisms responsible for the initiation and heterogeneous evolution of MM are poorly understood. Recent large-scale analyses of MM have focussed mainly on the protein-coding components of the genome, identifying recurrently mutated genes including *KRAS*, *NRAS*, *PRDM1*, *CCND1*, and *TP53* as drivers of tumourigenesis [1]. Many of these mutations are, however typically found at low frequency (<10% of tumours), and hence do not fully explain the clinical and biological diversity of MM. With the increasing availability of MM whole-genome sequencing (WGS) initiatives such as The Relating Clinical Outcomes in Multiple Myeloma to Personal Assessment of Genetic Profile Study (CoMMpass), we have sought to systematically search for driver mutations in the MM non-coding as well as coding regions of the genome. In our recent study, we reported an integrated analysis of the WGS of 765 and whole-exome sequencing (WES) of 804 MM patients from CoMMpass, identifying novel non-coding drivers altering expression of target genes as well as coding drivers [2]. We also demonstrated that pathways central to MM tumourigenesis could be targeted by both coding and non-coding drivers, further enhancing our understanding of alternative oncogenic pathways driving MM.

To search for non-coding drivers, we first defined the *cis*-regulatory elements (CREs) and promoters using information from promoter capture Hi-C in naïve B-cells [3] and transcription start site (TSS) proximity respectively. The approach enabled us to narrow down the genomic searches and mitigate against the high statistical burden in establishing significantly mutated regions. We identified promoters associated with 34 genes and CREs associated with 271 genes and as recurrently mutated by single nucleotide variants (SNVs). Many of the target genes are enriched for established oncogenic pathways in MM such as *PAX5* and *BCL6* in B-cell differentiation. Recurrent mutation of the *NBPF1* promoter corresponded to 1.7-fold increase in gene expression of *NBPF1*. Mutations in CREs of six target genes (*PAX5*, *ST6GAL1*, *COBLL1*, *HOXB3*, and *ATP13A2*) were also associated with differential gene expression. Notably, mutations in the *PAX5* CRE identified resulted in a 4.6-fold reduced expression, consistent with *PAX5* being a tumour suppressor as in other B-cell malignancies [4-6]. In contrast, disruption of *ST6GAL1* CRE by SNVs led to a 1.4-fold upregulation, consistent with *ST6GAL1* is overexpressed in various cancers [7] and aberrant glycosylation in MM [8].

We also found copy number variations at CREs regulate expression of seven candidate genes (*MYC*, *PACS2*, *TEX22*, *KDM3B*, *RAB36*, *PLD4*, and *SP110*). Notably, *MYC* oncogene was overexpressed in tumours having either deletion of upstream putative silencers or amplification of downstream putative enhancers. The amplified enhancers coincides with the *Myc* enhancer cluster essential for *MLL–AF9-driven* leukaemia in mice [9]. Our results demonstrate that *MYC* can be amplified via dysregulation of non-coding regulatory regions, in addition to the well-established mechanisms of *MYC* upregulation through chromosomal translocations and gene amplification.

To better understand the interplay of alternative somatic mechanisms underlying MM, we extended our analysis to categorise chromosomal copy number alterations, structural variations (SVs), and protein-coding drivers in CoMMpass dataset. We observed aberrant copy number alterations characteristic of MM [1]: gain of odd number chromosomes (in 59% tumours); deletions at 13q (63%), 14q (43%), 16q (38%), and 8p (38%); and amplification overlapping *MYC* and *PVT1* at 8q24.21. We detected novel SVs affecting genes including inversions disrupting *CYLD, MYC* translocations disrupting *CD96*, translocations intergenic to *PRDM1* and *FBXW7*, and translocations associated with upregulation of *MAP3K14* (7-fold) and *CCND2* (12-fold). We also identified 40 significantly mutated coding genes, 11 of which are novel (*BAX*, *C8orf86*, *FAM154B*, *FTL*, *HIST1H4H*, *LEMD2*, *PABPC1*, *RPN1*, *RPS3A*, *SGPP1*, *TBC1D29*).

**Figure 1:**
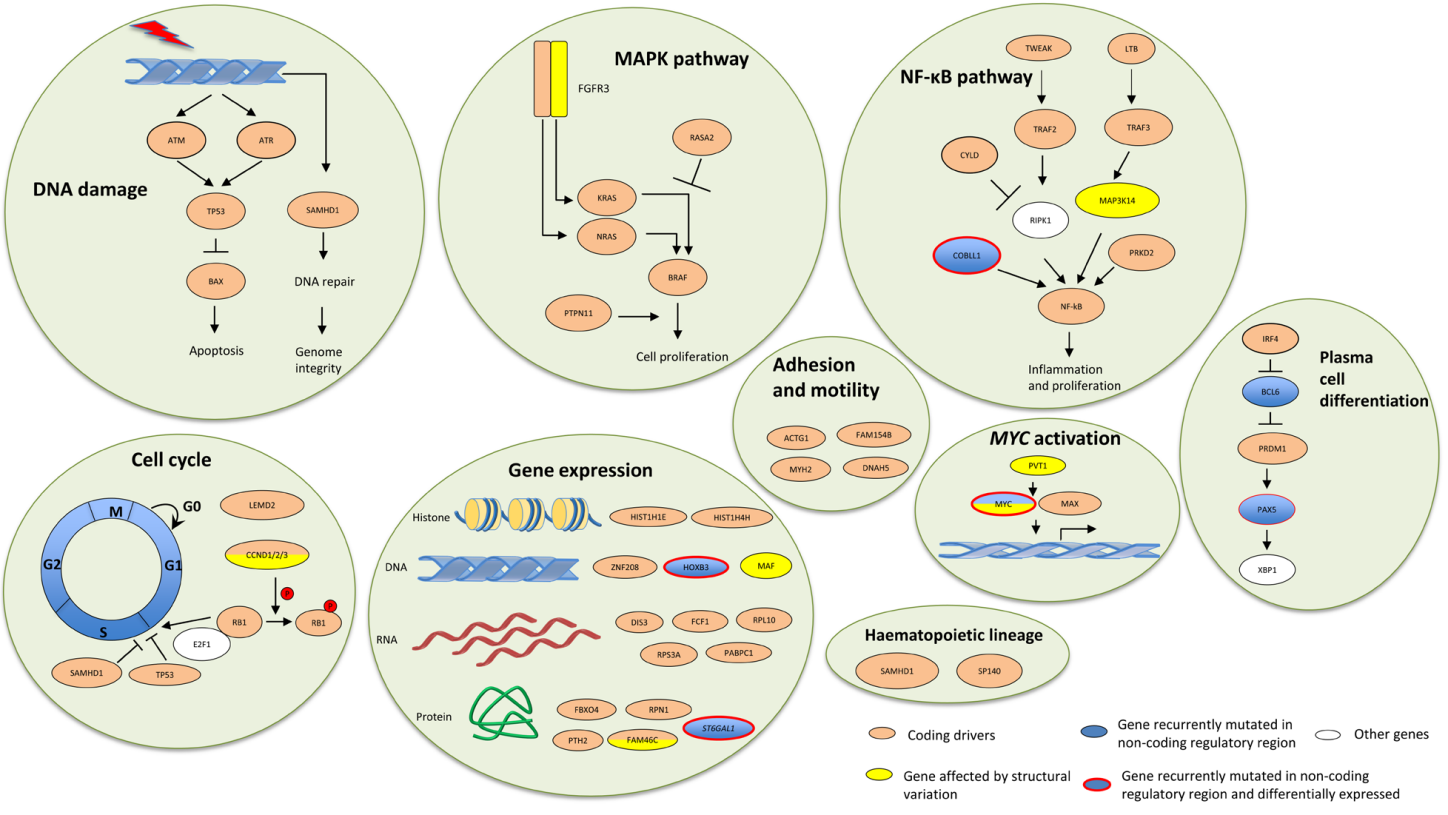
Key oncological pathways in multiple myeloma can be targeted by both coding and non-coding mechanisms Figure adapted from Hoang *et al* [2].

Performing an integrated analysis, allowed us to identify several key pathways somatically targeted by both coding and non-coding mutations (Figure 1). This is exemplified by plasma cell differentiation pathway in which we identified significant non-coding mutations associated with *BCL6* and *PAX5*, complementary to established coding drivers affecting *IRF4* and *PRDM1*.

Following on from these analyses, to gain insight into aetiological basis of somatic mutations in MM, we examined mutational signatures. Our findings are consistent with an association between the APOBEC family of cytidine deaminases signature 2 with coding drivers in *DNAH5*, *SAMHD1*, *TP53*, and *BRAF* along with and myeloma subtypes – t(14;16), t(14;20), and t(4;14). Additionally, we noted, AID-related signature 9 was more prevalent in MM than previously described (present in 96% of samples) and is associated with mutation affecting *PAX5* CREs. Finally, we also identified novel mutational signatures reflective of homologous recombination deficiency (signature 3 and 8) and of unknown aetiologies (signature 16 and 30) in >30% of tumours.

In summary, through identifying novel mutations disrupting coding and non-coding genomes, we have demonstrated that key biological pathways in MM could be targeted by alternative mechanisms. Our study thus further delineates the genomic complexity and heterogeneity underlying the disease, providing important information for potential development of novel therapeutic agents and offering perspectives for personalised therapy.
